# Breaking sarcomeres by *in vitro* exercise

**DOI:** 10.1038/srep19614

**Published:** 2016-01-25

**Authors:** Zacharias Orfanos, Markus P. O. Gödderz, Ekaterina Soroka, Tobias Gödderz, Anastasia Rumyantseva, Peter F. M. van der Ven, Thomas J. Hawke, Dieter O. Fürst

**Affiliations:** 1 Institute for Cell Biology, University of Bonn, Ulrich-Haberland-Str. 61a, 53121 Bonn, Germany; 2 Department of Pathology and Molecular Medicine, McMaster University, Hamilton, ON, L8N 3Z5, Canada

## Abstract

Eccentric exercise leads to focal disruptions in the myofibrils, referred to as “lesions”. These structures are thought to contribute to the post-exercise muscle weakness, and to represent areas of mechanical damage and/or remodelling. Lesions have been investigated in human biopsies and animal samples after exercise. However, this approach does not examine the mechanisms behind lesion formation, or their behaviour during contraction. To circumvent this, we used electrical pulse stimulation (EPS) to simulate exercise in C2C12 myotubes, combined with live microscopy. EPS application led to the formation of sarcomeric lesions in the myotubes, resembling those seen in exercised mice, increasing in number with the time of application or stimulation intensity. Furthermore, transfection with an EGFP-tagged version of the lesion and Z-disc marker filamin-C allowed us to observe the formation of lesions using live cell imaging. Finally, using the same technique we studied the behaviour of these structures during contraction, and observed them to be passively stretching. This passive behaviour supports the hypothesis that lesions contribute to the post-exercise muscle weakness, protecting against further damage. We conclude that EPS can be reliably used as a model for the induction and study of sarcomeric lesions in myotubes *in vitro*.

Skeletal muscle is plastic and adaptable, increasing in size with demanding exercise and decreasing with immobilisation, or during aging and disease^1^,^2^,^3^. Such size adaptations translate to changes in fibre size, and therefore sarcomere number, during which the muscle needs to maintain its functionality. The molecular events behind such remodelling on the sarcomeric level remain unclear.

Eccentric exercise, which triggers muscle hypertrophy^1^,^2^,^4^, leads to focal disruptions in the regular pattern of the myofibrils^5^,^6^. These disruptions, frequently referred to as sarcomeric “lesions”, were initially described in electron microscopy as electron-dense material “streaming” across adjacent Z-discs^5^,^6^,^7^. Lesions were envisioned as damage caused by eccentric contractions, due to the forceful lengthening of sarcomeres during their activation, as evidenced by a higher lesion prevalence after eccentric rather than concentric contractions in both animal models and humans^6^,^8^,^9^,^10^,^11^. Furthermore, the well-documented post-exercise loss of force was attributed to lesion formation^11^,^12^. This was supported by studies finding lesions but no other possible explanations for the weakness (such as sarcolemmal damage or immune cell infiltration) in post-exercised muscles^5^,^7^,^13^,^14^. In contrast, other studies found sarcolemmal damage post-exercise^15^,^16^, and the suggestion that the prevalence of lesions after exercise is not proportional to the observed force-reduction was put forth^17^. Instead, disruption of the excitation-contraction coupling or the extracellular matrix, fibre necrosis or inflammation were suggested to contribute to the muscle weakness^18^,^19^,^20^,^21^,^22^. Lesions were proposed to represent areas of remodelling^23^,^24^,^25^,^26^, potentially as a hypertrophic response to exercise. This is supported by evidence suggesting that lesions can escalate in size with time, and that young and old lesions differ in their immunopositivity for desmin and other specific sarcomeric proteins^23^,^24^,^27^. The precise function of lesions and the molecular mechanisms behind their formation and repair remain unclear. An obstacle is that these processes have mainly been studied in sections of post-exercised muscle samples, an approach which cannot address the dynamic formation of the lesions, or their behaviour during muscle activity.

Electrical pulse stimulation (EPS) has been applied *in vitro*, to stimulate contraction in myotubes. Most significantly, studies showed that EPS assists myotube differentiation by inducing sarcomere assembly^28^,^29^ and therefore the technology has been used for tissue engineering of both skeletal^30^,^31^,^32^,^33^,^34^,^35^,^36^ and cardiac^37^,^38^ muscle tissue constructs. EPS has also been used to simulate exercise in cultured myotubes, and to study its effects on mouse^39^,^40^,^41^,^42^,^43^,^44^,^45^,^46^ and human cells^47^ using markers such as increased glucose metabolism^40^,^41^,^47^, Akt phosphorylation^41^, upregulation of exercise-related chemokines^42^,^43^,^44^,^45^ and induction of Hsp70 expression^46^. Overall, these studies show that EPS can be effectively used to model exercise *in vitro*.

Recently, while applying EPS on mouse myotubes for the purposes of exercise simulation, we noticed the appearance of structures that we identified to be sarcomeric lesions positive for the “lesion markers” filamin-C (FLN-C) and Xin^48^. In the current work, in order to further characterize this phenomenon we tested different EPS protocols and noticed an increased incidence of lesion formation with time and EPS intensity. Furthermore, using transfections of an EGFP-tagged marker we observed the formation of these lesions, as well as their behaviour during contraction, by live microscopy. We conclude that EPS is a powerful tool for the induction of sarcomeric damage in cultured myotubes, and its analysis in living cells.

## Results

### EPS-induced *in vitro* exercise leads to the formation of sarcomeric lesions

For our EPS experiments we used differentiated myotubes grown on coverslips, derived from C2C12 myoblasts of a low passage number. Application of short electrical pulses on the myotubes resulted in twitch contractions, whereas application of multiple pulses at a high frequency (15 Hz) resulted in fused tetanus, which relaxed immediately after the stimulation was terminated. The responsiveness of the myotubes to the EPS allowed us to test different contraction protocols, two of which were used to induce sarcomeric lesions (see Methods, and Supplementary Fig. S1). The milder “Twitch” protocol induced one brief twitch contraction per second. The “Damage” protocol aimed at a stronger effect, and induced an alternation between very fast twitches and a tetanic hold. Both protocols were applied for up to 5 hrs, while coverslips were sampled at regular intervals.

**Figure 1:**
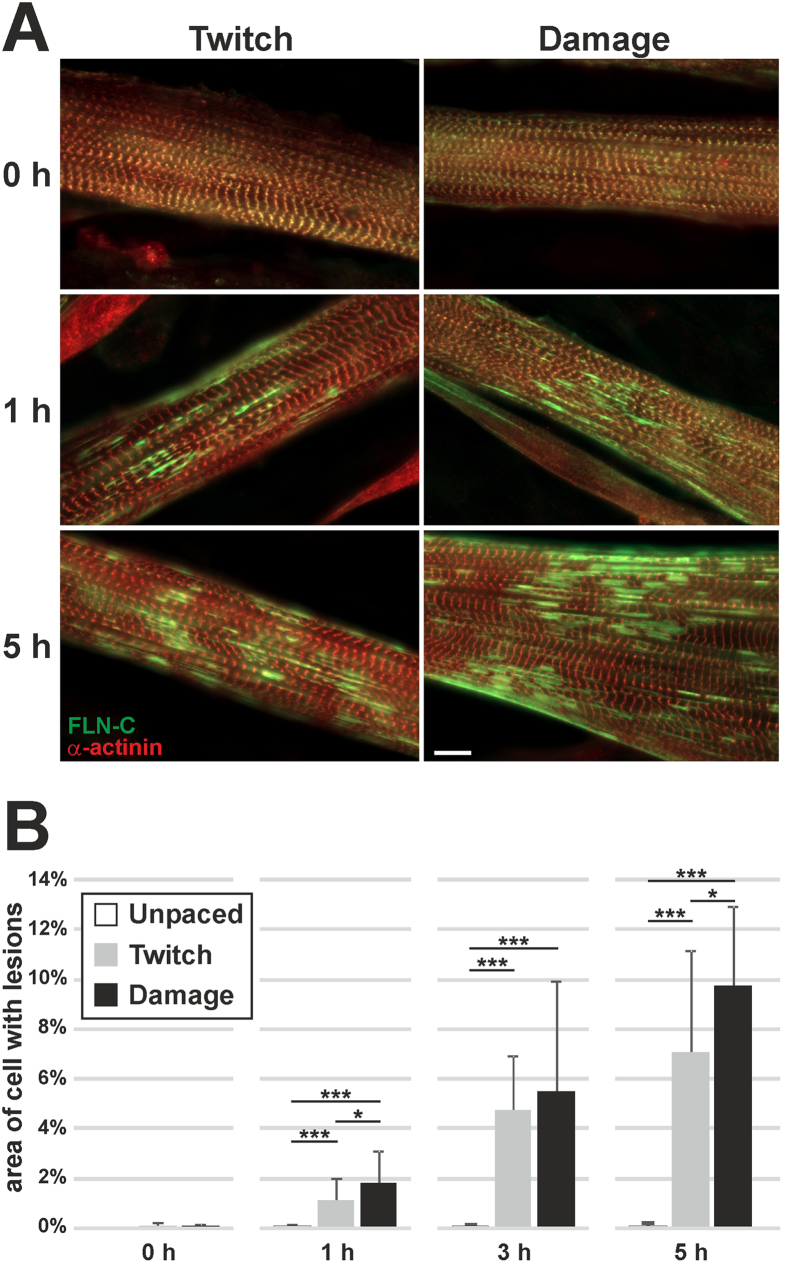
Application of two EPS protocols on C2C12 myotubes leads to the formation of sarcomeric lesions, increasing with time. (**A**) Representative images of myotubes treated with EPS, stained for the Z-disc marker α-actinin and the lesion marker FLN-C. The incidence of lesions increases with time of pacing. Note that even before pacing, the myotubes have fully formed and laterally aligned sarcomeres. (**B**) Quantification of lesion formation, represented as lesion area normalised to the cell area within each photograph (20 cells photographed per timepoint per treatment). Lesion area significantly increases with pacing, whereas the unpaced controls remain unchanged with time. The damage protocol has a significantly stronger effect than the twitch protocol (error bars represent standard deviation, significance by student’s t-test). (Scale bar = 10 μm).

Staining with antibodies specific for the Z-disc markers α-actinin and FLN-C showed that the myotubes, even before any EPS treatment, had fully-formed and laterally-aligned Z-discs (Fig. 1A). Upon application of both EPS protocols however, FLN-C positive sarcomeric lesions began to appear, increasing in number with time (Fig. 1A). In order to quantify the appearance of the lesions, multiple images of myotubes from separate experiments were taken according to specific criteria (see Methods and Supplementary Fig. S2) by fluorescence microscopy (20 cells per timepoint per treatment). The percentage of the myotube area covered by lesions at each timepoint was calculated (Fig. 1B). Even after only 1 hr of EPS, a significant prevalence of lesions was seen using both protocols, progressively increasing with time. Furthermore, despite the high variability observed, the Damage protocol was significantly more effective in inducing lesions than the Twitch protocol. Altogether, exercise mimicking by EPS on C2C12 myotubes induces sarcomeric lesions, with prevalence increasing with time or intensity.

**Figure 2:**
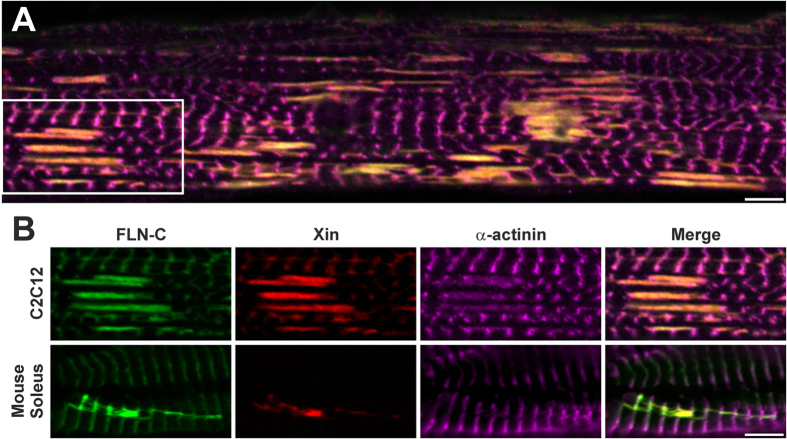
EPS-induced lesions in C2C12 cultured myotubes resemble those seen after downhill running in mouse soleus muscles. (**A**) Confocal section of a myotube after 5 hrs of damage EPS stained for the lesion markers Xin and FLN-C shows the colocalisation of those markers, and the morphology of the lesions forming between adjacent Z-discs. (**B**) Lesions from the soleus muscle of a downhill-run mouse appear similar to the EPS-induced lesions in myotubes (part of previous panel). (Scale bars = 5 μm).

In order to further characterise the EPS-induced lesions, we stained myotubes grown on coverslips and treated with the Damage protocol for 5 hrs, for the sarcomeric damage marker Xin (Fig. 2A). Confocal microscopy showed that in all cases lesions extended from Z-disc to Z-disc, spanning one or more sarcomeres. Xin colocalised with FLN-C in the lesions, whereas only a weak α-actinin signal could be seen in these structures. We then compared the EPS-induced lesions in the myotubes with lesions induced *in vivo* by eccentric exercise, in soleus muscles obtained from mice that had undergone downhill treadmill running. Confocal microscopy using the same markers showed that the lesions in the cultured myotubes and the muscle fibres of the exercised mice were highly similar, and that FLN-C colocalised with Xin in all lesions (Fig. 2B).

### Lesions form progressively over time

**Figure 3:**
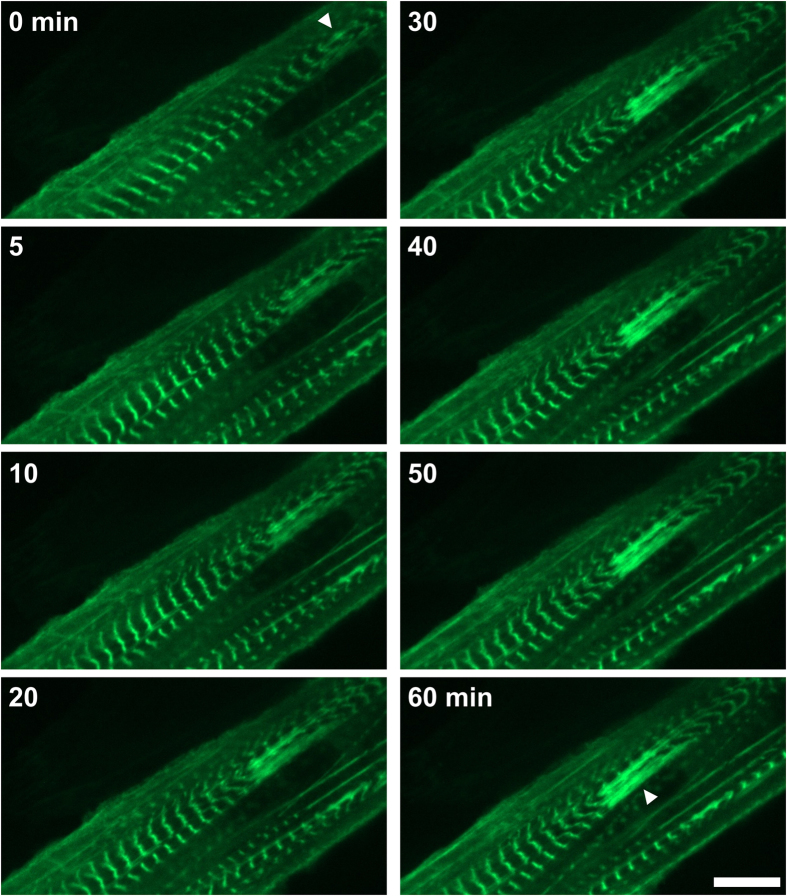
Lesion formation seen by live cell imaging of C2C12 expressing FLNc-EGFP. Cells were periodically photographed during damaging EPS. Note that the lesion progressively grows, while the fluorescence of FLNc-EGFP increases in the lesion. (Scale bar = 10 μm).

Despite the multiple reports of sarcomeric lesions in the literature, it is still unclear how these structures arise and develop over time. In order to address this, we performed live experiments in C2C12 myotubes transiently expressing human filamin C (FLNc) fused to EGFP (FLNc-EGFP). FLNc was chosen as it localises both in the Z-discs and lesions, and is therefore an ideal marker for such work. The protein localised at the Z-discs of transfected cells, and pacing allowed the visualisation of the individual Z-discs during contraction using spinning disk confocal microscopy. Cells were observed during application of the Damage protocol. Periodically, EPS was briefly paused to allow imaging. Figure 3 shows the progressive development of a lesion (arrow head). The structure was initiated by FLNc-EGFP “streaming”, spanning a couple of sarcomeres. Progressively the lesion grew, extending laterally and involving more sarcomeres. Similar results were obtained using primary mouse myoblasts (Supplementary Fig. S3). The experiments showed that in this model lesions form progressively, over a period of tens of minutes, in response to EPS.

### Lesions display passive elastic-like behaviour during contractility

**Figure 4:**
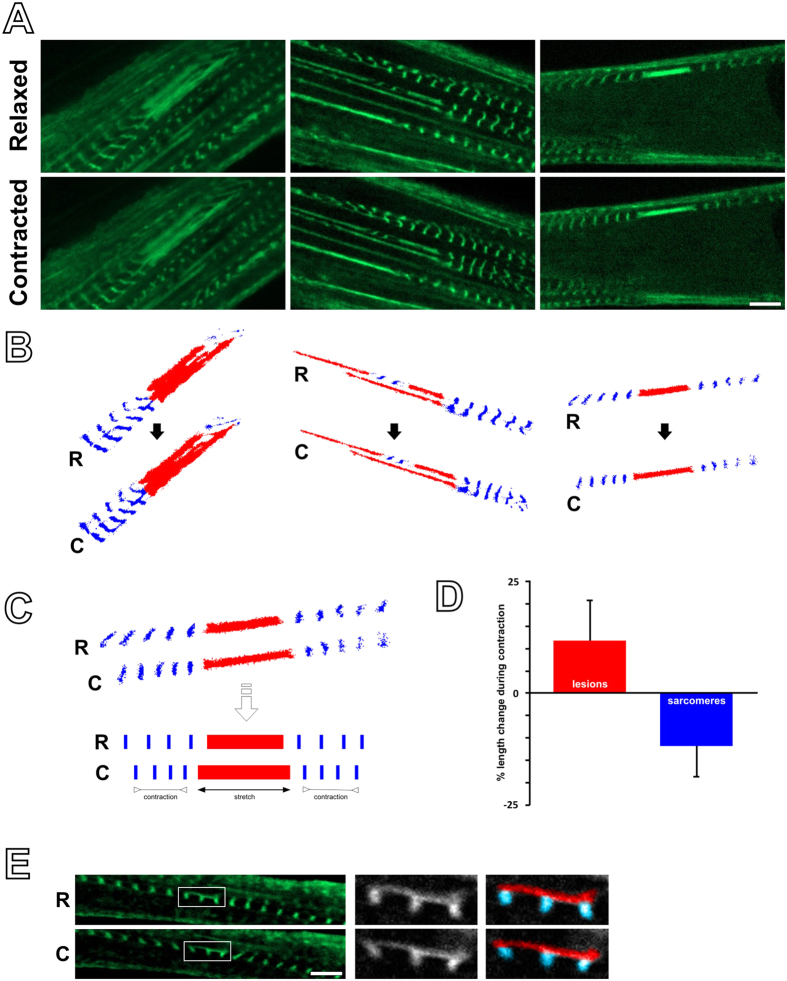
Lesions stretch during contraction. (**A**) Examples of lesions photographed in a relaxed state and during a tetanic contraction, as seen by live cell imaging in C2C12 myotubes expressing FLNc-EGFP. (**B**) For clarity, lesions from the panels above are marked in red, and surrounding Z-discs of the neighbouring sarcomeres in blue. (**C**) During contractions, lesions become stretched and elongate, whereas the neighbouring sarcomeres shorten (as seen by their Z-discs). (**E**) Quantification of the percentage change in length of the lesions and neighbouring sarcomeres (lesions from 23 individual cells analysed, error bars represent standard deviation). (**F**) Example of two sarcomeres showing Z-disc streaming only in part; during contraction, the part with the lesion stretches, while the unaffected part contracts. (Scale bar = 5 μm).

We then studied the behaviour of the lesions during contraction. Multiple lesions were imaged during a tetanic contraction, versus during relaxation (Fig. 4A). The length of the lesions as well as the length of the surrounding sarcomeres were measured (Fig. 4B). During contraction, sarcomeres shortened as expected, whereas lesions were seen to lengthen (Fig. 4C,D). There were also rare cases, where FLNc-EGFP streaming was seen only within part of a single myofibril (Fig. 4E). Even there, during tetanic contraction the affected part of the sarcomere displaying streaming lengthened, whereas the unaffected part contracted. Altogether this shows that not only lesions are non-contractile, but may also be introducing “slack” to the affected myofibrils, by showing elastic-like behaviour during contraction.

## Discussion

In this work we present the application of the EPS technology to study the formation and function of sarcomeric lesions in skeletal muscle cells. We show that EPS can induce those structures in cultured myotubes, proportionally to the amount of time of EPS application and protocol intensity. Using FLNc-EGFP transfections we demonstrate that lesion formation, as well as their behaviour during contractility, can be followed using live cell imaging.

Lesions are known to form mainly after eccentric rather than concentric contractions^6^,^9^,^10^, even though Z-disc disruption has also been reported after concentric exercise induced by electrical stimulation^49^. Our cell model is not eccentric, as the myotubes are not extending while contracting, and have a low range of motion. It is also not entirely concentric as they are attached on the substrate, and therefore do not shorten across their entire length during contraction. Culturing cells on elastic substrates, thus permitting concentric contraction, could circumvent this limitation. Eccentric contraction could be simulated by combining EPS on elastic substrates with stretching. Stretching alone has been successfully used to induce lesions in cardiomyocytes^50^, although we are unaware of such a perspective on cells of skeletal muscle origin.

The application of EPS combined with live microscopy adds the advantage of being able to observe the formation of lesions as they emerge, something not possible with post-exercise sampling in man and in animal models. Here we observed that under continuous EPS lesions started as small focal streaming and increased in size with time. Furthermore, filamin-C intensity was stronger in the lesions both by immunostaining and transfection during live experiments. A progression in lesion formation has been proposed before. Thompson *et al*.^27^ observed different sizes of lesions, displaying different patterns under toluidine blue staining. They reasoned that larger lesions may gradually progress from smaller lesions. Subsequently, Yu *et al*.^23^,^24^ showed that some lesions stained positive for desmin, whereas others were negative. They proposed that this represents different ages of a lesion, with desmin staining initially disappearing, but returning as the remodelling process takes place, agreeing with older observations from other groups^11^,^51^. These studies were based on sampling different timepoints, rather than observing the dynamic formation and progression of a single lesion live. Although this is not possible in living animal models, live cell imaging coupled with simultaneous transfection of different constructs circumvents this. Future work should focus on investigating the timescale and proteins involved in lesion formation, as well as in repair.

Sarcomeric lesions have been suggested to be the reason behind the observed post-exercise muscle weakness^11^,^12^. A counter-argument is that in some studies, the muscle area occupied by lesions after exercise is very small compared to the overall strength loss^17^. This is a strong argument, provided that lesions represent nothing more than inactivated sarcomeres, no longer contributing to force generation. However, here we have shown that (at least in our model) lesions are not only non-contractile, but also stretchable. Their formation therefore, could introduce slack to the myofibrils, potentially reducing the overall tension or force transduction capability of the fibre. Furthermore, sarcomeres neighbouring to lesions may be working at smaller initial (pre-contraction) lengths, leading to a further decreased force output. Finally, desmin is absent around newly-formed lesions^23^,^24^, suggesting a weakness in the tension transmission, which may help explain the stretchability of lesions. Altogether, lesions may dissipate or absorb part of the work produced by the entire myofibril that they are found in. This may provide an explanation for the discrepancy between the strong effect in muscle weakness, and the relatively small amount of lesions seen.

One may speculate, that the added slack that the lesions provide during exercise may protect the muscle from further damage. It has been proposed that during eccentric exercise the reduction in force takes place gradually within the first few minutes of an exercise event^51^,^52^. This may reflect the time it takes for lesions to form, progressively relaxing the tension in the fibre. In the long-term, lesions disappear from the muscle fibres^53^,^54^, indicating repair of the damaged myofibrils. Remodelling processes may provide the muscle with more length, so that subsequent exercise would not cause further damage. This may explain the phenomenon known as the repeated bout effect, in which one exercise bout protects against damage from subsequent bouts^21^,^55^,^56^. Therefore, lesion formation may represent an adaptation with both short-term and long-term functions.

EPS has been used to mimic exercise *in vitro*^39^,^40^,^41^,^42^,^43^,^44^,^45^,^46^. In this work, we demonstrate that a known morphological marker of exercise, the formation of sarcomeric lesions, can also be seen in EPS-treated myotubes. The technique can also be applied on primary mouse cells, opening up the possibility of analysing myotube cultures derived from diseased or genetically modified mouse models. Most importantly, live cell imaging during EPS allows the study of the formation and behaviour of lesions, something that cannot be achieved in animal models.

## Methods

### Cell culture

C2C12 cells were proliferated in medium containing 15% FCS, 2 mM non-essential amino acids, 1 mM sodium pyruvate, 100 U/ml penicillin and 100 μg/ml streptomycin, and differentiated in medium containing 2% HS, 2 mM non-essential amino acids, 1 mM sodium pyruvate, 100 U/ml penicillin and 100 μg/ml streptomycin, both in Dulbecco’s modified Eagle medium (DMEM), high glucose, with GlutaMax (all components from Thermo Fischer Scientific Gibco, Darmstadt, Germany). Media were changed every two days. For EPS lesion induction and quantification, cells were seeded on ethanol-washed and autoclaved 15 mm coverslips placed in 6-well plates (CytoOne/Starlab, Hamburg, Germany). For live experiments, cells were seeded in 2 cm Lab-Tek chamber slides (Thermo Fischer Scientific Nunc, Rochester, USA) and transfected with a construct encoding human FLNc fused to EGFP^57^ using JetPrime (Polyplus-transfection SA, Illkirch, France) for 4–6 hours according to the instructions of the manufacturer, using 2 μg DNA and 4 μl JetPrime in 200 μl JetPrime buffer per well of a 6-well plate, or per 2 wells of a Lab-Tek chamber slide.

### Mouse treadmill exercise

All experimental protocols were approved by the McMaster University Animal Care Committee (AUP #09-08-29) in accordance with the Canadian Council for Animal Care guidelines. Male C57BL/6J wild-type mice were obtained from Jackson Laboratories (Bar Harbor, ME, USA). Animals were housed in a temperature- and humidity-controlled facility with a 12/12 h light/dark cycle and had ad libitum access to water and food. To induce physiologically relevant muscle damage, mice (3–5 months old) were run downhill on a 6M-Treadmill (Columbus Instruments, Columbus, state, USA) at a decline of 15° for 5 min at 5 m/min, to adapt them to the downhill running protocol, followed by 55 min at 10 m/min. Five hours following the exercise, the soleus muscles were harvested, embedded in optimal cutting compound and snap frozen in liquid nitrogen cooled isopentane.

### Electrical pulse stimulation

Cells were stimulated using a C-Pace unit and a 6-well C-Dish, according to the manufacturer (Ion Optix, Milton, MA, USA) applying pulses for a duration of 20 ms at 10 V. Two different frequency protocols were applied. The “twitch” protocol consisted of one pulse per second (1 Hz). The “damage” protocol consisted of a sequence of a 5-second tetanic hold effected by continuous pulses at 15 Hz, 5 seconds delay, 5 seconds of pulses at 5 Hz followed by another 5 seconds delay (the purpose of the delays was to allow for calcium store replenishment and myotube adhesion recovery). For live lesion formation experiments, the damage protocol was applied on cells seeded in a Lab-Tek chamber using homemade 1 mm-thick electrodes, 2 cm apart, with the pacing paused every 5 minutes for image recording. Tetanic contractions for the study of lesion behaviour during contraction were induced using continuous pulses of 15 Hz as above, and the same electrodes.

### Immunostaining

Cells on coverslips were fixed in a 1:1 mixture of methanol and acetone at -20 °C for 5 minutes and blocked in 10% normal goat serum and 1% BSA in PBS for 20 minutes. Primary antibodies (mouse monoclonal RR90, recognising FLNc and FLNa^58^; mouse monoclonal XR1, recognising XinA and XinB^59^; rabbit serum RaA653 against sarcomeric α-actinin^58^) diluted in PBS containing 0.05% Tween (PBST) were applied on the coverslips for 1 hr in a moist chamber. Coverslips were washed twice by immersion in PBST, incubated with secondary antibodies conjugated to FITC, Cy3 or Alexa Fluor 647 (Jackson ImmunoResearch/Dianova, Hamburg, Germany) for 30 minutes and washed as before in PBST and once in distilled water before mounting in Mowiol containing 10% N-propyl gallate. Tissue cryosections of soleus muscles from exercised mice on slides were fixed with methanol (2 min) followed by acetone (20 sec) at −20 °C and blocked as above for 30 minutes. Primary antibodies (as above) were applied in 1% BSA in PBS consecutively overnight at 4 °C, slides were washed twice in PBST and once in PBS (5 minutes each) and secondary antibodies (as above) were applied in 1% BSA in PBS consecutively at 37 °C for 2.5 hrs each. Slides were washed as before and gently rinsed in distilled water before mounting as above.

### Microscopy

For lesion quantification, myotubes fulfilling appropriate criteria (thicker than 10 μm, containing laterally-aligned sarcomeres) were randomly selected. In all cases, areas containing lesions were photographed avoiding areas in close proximity (<10 μm) of myotube branching. Images were recorded using a Zeiss Imager M1 fluorescence microscope and Zen pro 2012 (blue edition) (Carl Zeiss, Jena, Germany), using the auto-exposure settings, and processed for quantification as described in the next section.

Confocal images were recorded using a Zeiss LSM 710 and Zen 2012 (black edition) (Carl Zeiss). Live imaging was performed using a Zeiss Cell Observer SD spinning disk microscope and Zen 2012 (blue edition) (Carl Zeiss). For the study of lesion behaviour during contraction, videos of cells transfected with FLNc-EGFP were recorded while the cells were induced to contract tetanically by EPS (as described above) for a few seconds, starting from a relaxed state, thus recording both the relaxed and contracted states. Images of the relaxed and contracted states were exported from the videos and analysed as described in the next section.

### Imaging analysis

For lesion area calculations after EPS, lesion areas from each photograph taken were digitally quantified (see example in Supplementary Figure S2). FLN-C gave a stronger signal at the lesions than at the Z-discs, while conversely, the signal for α-actinin was strong in the Z-discs but very weak in the lesions. In order to optimise quantification of only the lesion signal, the α-actinin channel was digitally subtracted from the FLN-C channel, effectively removing the Z-disc signal (where the two channels are colocalising). This subtraction worked both in ImageJ^60^ (Process > Image Calculator, Subtraction), or the similar tools in Adobe Photoshop CC (2014, Adobe Systems, Dublin, Ireland). In order to process images in batch, the procedure was performed using the DOS-based program “subtract background” written in Haskell language, which further optimizes the subtraction procedure by testing different gain settings for each image (see Supplementary Material). Output images were processed in ImageJ or Adobe Photoshop CC by manually applying an intensity mask separating the lesion area from the remaining image and quantifying it in pixel number (in ImageJ, menus Image > Adjust > Threshold, and Analyse > Analyse Particles). The cell area in the image was manually marked and quantified in pixels (in ImageJ, menu Analyse > Measure). The lesion area value was divided to the cell area value and expressed in percentage. For each timepoint and treatment, 20 photographs (depicting one cell each) from 2 separate replicates (10 per replicate) were processed and pooled to derive the means (this is a total of 20 cells per timepoint per treatment).

For the experiments on lesion behaviour during contractility (Fig. 4D), the percentage change in length of sarcomeres and lesions between a relaxed and a contracted state were quantified. In total, 23 sets of photographs, each set showing a different cell in a relaxed and a contracted state, were processed (total of 23 cells) in Zen 2012 (blue edition, Carl Zeiss) for measurements. For each set, the length of a lesion in both states was measured, and the change was expressed in percentage. Also for each set, the total length of 3 consecutive sarcomeres (to minimise human error in measurement) on either side of the lesion (skipping the one sarcomere immediately left or right to the lesion to avoid measuring damaged sarcomeres) was measured in both states, and the percent change calculated (the measurements were taken from both sides of the lesion to ensure that sarcomeres were still functional at both locations). This gave rise to a total of 23 measurements of lesion-length change and 46 measurements of sarcomere triplet length change. The measurements of each of the two structures were respectively pooled to derive the mean percent change.

## Additional Information

**How to cite this article**: Orfanos, Z. *et al*. Breaking sarcomeres by *in vitro* exercise. *Sci. Rep*. **6**, 19614; doi: 10.1038/srep19614 (2016).

## Supplementary Material

Supplementary Information
