# Supplementary material for: Breaking sarcomeres by in vitro exercise
Source: Sci Rep. 2016 Jan 25;6:19614. doi: 10.1038/srep19614 (PMC4726327; doi:10.1038/srep19614)
Supplement: Supplementary Information [file srep19614-s1.pdf]

Supplementary Material

Main article: Breaking sarcomeres by *in vitro* exercise, by Zacharias Orfanos, Markus P. O. Gödderz, Ekaterina Soroka, Tobias Gödderz, Anastasia Rumyantseva, Peter F. M. van der Ven, Thomas J. Hawke and Dieter O. Fürst

Supplementary Figures

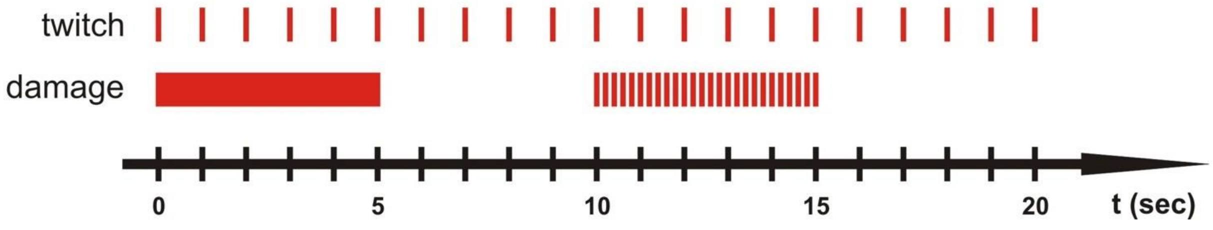

Supplementary Figure S1. Schematic representation of the two EPS protocols used in this study. Red lines indicate single 20 msec twitches, whereas the filled bar in the damage protocol represents continuous stimulation (by a sequence of a very fast succession of 20 msec twitches at 15 Hz) for the indicated time. After the 20 seconds represented here the programs are repeated.

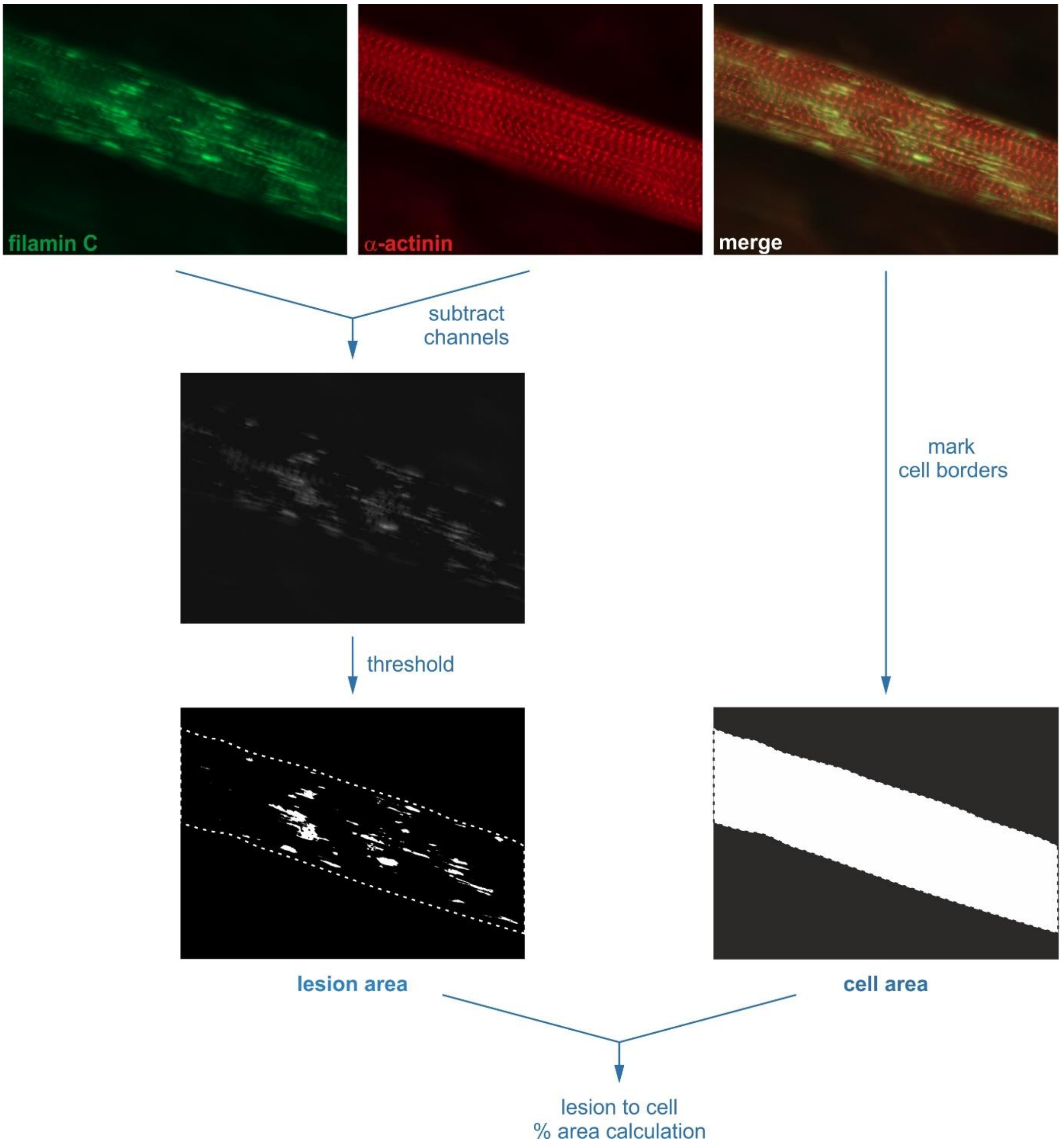

Supplementary Figure S2. Description of imaging analysis for lesion area calculation. The α-actinin channel was mathematically subtracted from the filamin C channel, to minimise the Z-disc signal and focus only on the lesions. The resulting image was manually "thresholded", and the area of lesions calculated digitally. The area of the cell in the image was manually marked and digitally calculated. The two measurements yield the percentage of the cell area in the image occupied by lesions.

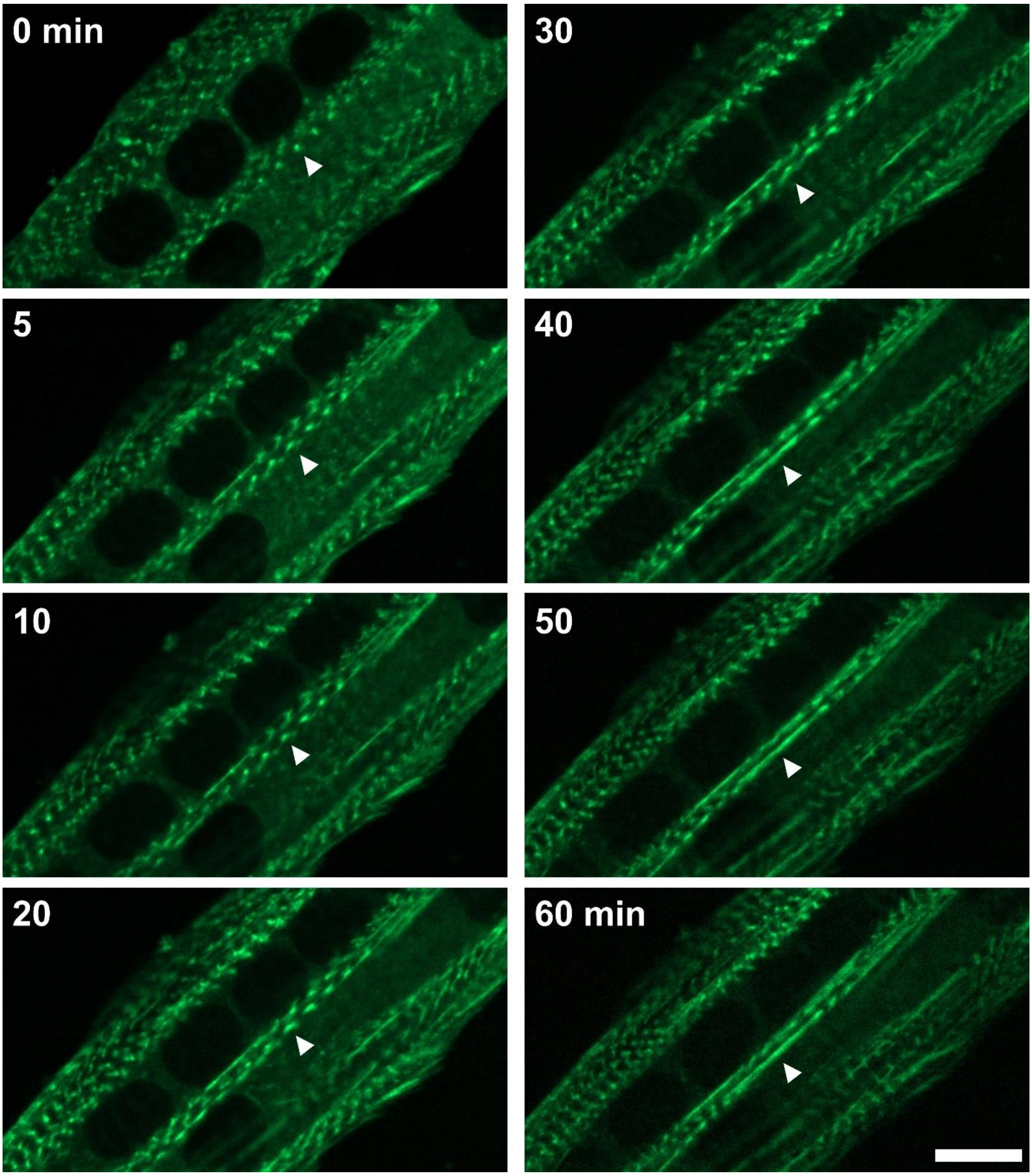

Supplementary Figure S3. Lesion formation observed by live cell imaging of primary mouse myoblasts expressing FLNc-EGFP. Cells were periodically photographed during damaging EPS. Note that the Z-discs begin to widen before streaming is seen at 40 mins after application. (Scale bar = 10 μm)

Supplementary Method

Program for subtracting the α-actinin channel image from the FlnC channel image

For the lesion area quantification described in the main Methods, we subtracted the α-actinin channel image from the FlnC channel image. The majority of the Z-disc signal was eliminated in the resulting image, permitting an easier subsequent application of the intensity threshold mask on the remaining lesion areas for quantification. This subtraction works well manually in both ImageJ and Photoshop, however in order to process images in batch, we wrote a program. The program further optimized the images for subtraction by adjusting the intensity of the α-actinin channel image before subtraction, bringing both images to a similar average intensity. This was done to avoid artefacts that could result from this subtraction, as a consequence of the unavoidable overall differences in intensity between the different channels during acquisition.

In the program, the background image *B* (α-actinin channel) is subtracted from the foreground image *A* (FlnC channel) as follows: the luminance of the background image is scaled by a factor *f*. This factor is chosen so that it minimizes the average difference in luminance of pixels at the same coordinate. More precisely: Let *I* be the set of coordinates of both images—assuming they both have the same resolution—and let $(a_i)_{i \in I}$ and $(b_i)_{i \in I}$ be the luminance values of pixels in *A* and *B*, respectively. Then *f* satisfies:

$$\min_{f \in R} \sum_{i \in I} |a_i - fb_i|.$$

The new image *C* results by subtracting the luminance-adjusted *fB* from *A*, pixel-wise:

$$c_i := \max\{0, a_i - fb_i\} \quad (\forall i \in I)$$

This procedure is invariant under the exposure of *B*—as long as it is not under- or overexposed—which makes it useful for reproducible results. The minimum of linear distances (rather than quadratic, for example) in the definition of *f* was chosen because it reduces over-compensation due to significantly brighter parts in *A*—the parts that should be revealed.

Source Code follows on next page.

```
import Codec.Picture
import Codec.Picture.Types
import Control.Monad
import Control.Parallel.Strategies
import Data.Either.Combinators
import Data.List
import Debug.Trace
import System.Environment
import System.FilePath
import System.FilePath.Glob
import System.Exit
import qualified System.Info

type DefPixel = Pixel16
type DefImage = Image DefPixel
createDefImage = ImageY16

-- Missing:
-- CMYK8, CMYK16, YCbCr8, F, RGBF
toDefImage :: DynamicImage -> DefImage
toDefImage (ImageY16 img)    = img
toDefImage (ImageY8 img)     = pixelMap promotePixel img
toDefImage (ImageYA8 img)    = pixelMap (promotePixel . dropTransparency)
img
toDefImage (ImageYA16 img)   = pixelMap dropTransparency img
toDefImage (ImageRGB8 img)   = pixelMap (promotePixel . compLuma) img
toDefImage (ImageRGBA8 img)  = pixelMap (promotePixel . compLuma .
dropTransparency) img
toDefImage (ImageRGB16 img)  = pixelMap compLuma img
toDefImage (ImageRGBA16 img) = pixelMap (compLuma . dropTransparency) img
toDefImage _ = error "Unhandled image type"

-- Override some computeLuma instances for more efficiency: Rationals are
slow.



genericRbgLuma r g b = floor $ 0.3 * fromIntegral r +
                               0.59 * fromIntegral g +
```

```
                              0.11 * fromIntegral b

class MyLuminizable a where
    compLuma :: a -> PixelBaseComponent a

instance MyLuminizable PixelRGB16 where
    compLuma (PixelRGB16 r g b) = genericRbgLuma r g b

instance MyLuminizable PixelRGBA8 where
    compLuma (PixelRGBA8 r g b _) = genericRbgLuma r g b

instance MyLuminizable PixelRGB8 where
    compLuma (PixelRGB8 r g b) = genericRbgLuma r g b

halveList list = (fsts, snds)
    where
        half = length list `div` 2
        fsts = take half list
        snds = drop half list

makePairs :: [a] -> Either String [(a, a)]
makePairs xs = if even (length xs)
               then Right $ uncurry zip $ halveList xs
               else Left "Odd number of arguments."

errExit :: String -> IO a
errExit err = usage >> putStrLn err >> exitFailure

orAbort :: Either String a -> IO a
orAbort (Left err) = errExit err
orAbort (Right x)  = return x

usage :: IO ()
usage = do
    progName <- getProgName
    putStrLn $ progName ++ " foreground1 [foreground2 ...] background1
[background2 ...]"
```

```
readImages :: [String] -> IO (Either String [DefImage])
readImages = liftM sequence . mapM (liftM (mapRight toDefImage) .
readImage)

totalBrightness :: DefImage -> Integer
totalBrightness img = sum [ fromIntegral (brightnessAt img x y)
                          | x <- [0..width-1]
                          , y <- [0..height-1]
                          ]
    where width  = imageWidth img
          height = imageHeight img

subtractImage :: (DefImage, DefImage) -> Either String DefImage
subtractImage (img1, img2) =
    let maxFac  = fromIntegral (totalBrightness img1) / fromIntegral
(totalBrightness img2)
        fac     = optimize (linearCost img1 img2) 0 maxFac
        width   = imageWidth img1
        width2  = imageWidth img2
        height  = imageHeight img1
        height2 = imageHeight img2
    in if width == width2 && height == height2
       then Right $ generateImage (newPixies fac img1 img2) width height
       else Left "Image Dimensions don't match."

--cm :: Monad m => (a -> m [b]) -> [a] -> m [b]
--cm f xs = liftM concat $ map f xs

concatMapM f = liftM concat . mapM f

parallelList = withStrategy (parList rdeepseq)

main = do
    filePaths <- if "mingw" `isPrefixOf` System.Info.os
                 then getArgs >>= concatMapM namesMatching
                 else getArgs
    images <- readImages filePaths
```

```
    imagePairs <- orAbort $ images >>= makePairs
    when (null imagePairs) (errExit "No arguments given.")
    resultImages <- orAbort $ sequence $ parallelList $ map subtractImage
imagePairs
    let newNames = map (flip replaceExtension ".out.png") filePaths
    mapM (uncurry savePngImage)
         (zip newNames
              (map createDefImage resultImages)
         )

sub :: Pixel16 -> Pixel16 -> Pixel16
sub p1 p2 = if p1 >= p2 then p1 - p2 else 0

mult :: Float -> DefPixel -> DefPixel
mult f p = m p
    where m = round . (f*) . fromIntegral

newPixies :: Float -> DefImage -> DefImage -> Int -> Int -> DefPixel
newPixies fac img1 img2 x y = rv
    where p1 = pixelAt img1 x y
          p2 = pixelAt img2 x y
          rv = p1 `sub` (fac `mult` p2)

brightnessAt :: DefImage -> Int -> Int -> Int
brightnessAt img x y = fromIntegral (pixelAt img x y)

quadraticCost img1 img2 f = sum $ map square $
        [ fromIntegral (brightnessAt img1 x y)
          - f * fromIntegral (brightnessAt img2 x y)
        | x <- [0..width-1]
        , y <- [0..height-1]
        ]
    where width  = imageWidth img1
          height = imageHeight img1
          square x = x * x

linearCost img1 img2 f = sum $ map abs $
```

```
        [ fromIntegral (brightnessAt img1 x y)
          - f * fromIntegral (brightnessAt img2 x y)
        | x <- [0..width-1]
        , y <- [0..height-1]
        ]
    where width  = imageWidth img1
          height = imageHeight img1

optimize :: (Float -> Float) -> Float -> Float -> Float
optimize cost lo hi = optimize' cost lo hi (cost lo, cost mid, cost hi)
    where mid = (lo + hi) / 2

-- Precision of optimize/optimize' (x-wise)
eps = 0.02

optimize' :: (Float -> Float) -> Float -> Float -> (Float, Float, Float) ->
Float
optimize' _ lo hi _ | hi - lo < eps = (lo + hi) / 2
optimize' cost lo hi (ylo, ymid, yhi) =
    let xs = map (\i -> lo+i*(hi-lo)/4) [0..4]
        ys = [ylo, cost (xs !! 1), ymid, cost (xs !! 3), yhi]
        maxY = minimum ys
        maxI = head $ filter ((maxY ==) . (ys !!)) [0..4]
        loI = max 0 (maxI - 1)
        hiI = min 4 (maxI + 1)
    in optimize' cost (xs !! loI) (xs !! hiI) (ys !! loI, ys !! (loI + 1),
ys !! hiI)

opt' :: (Float -> Float) -> Float -> Float -> Float
opt' cost lo hi =
    let n = 20
        xs = map (\i -> lo+i*(hi-lo)/fromIntegral n) [0 .. fromIntegral n]
        ys = map cost xs
        maxY = minimum ys
        maxI = head $ filter ((maxY ==) . (ys !!)) [0..n]
    in trace (concatMap ((++"\n").show)$ zip xs ys)
    $ xs !! maxI
```
